# MULTIDIMENSIONAL CIVIC ENGAGEMENT OF OLDER PEOPLE: A LATENT CLASS ANALYSIS

**DOI:** 10.1093/geroni/igad104.1673

**Published:** 2023-12-21

**Authors:** Toon Vercauteren, Marina Näsman, Fredrica Nyqvist, Dorien Brosens, Rodrigo Serrat, Dury Sarah

**Affiliations:** Vrije Universiteit Brussel, Brussels, Brussels Hoofdstedelijk Gewest, Belgium; Åbo Akademi University, Vaasa, Pohjanmaa, Finland; Åbo Akademi University, Vaasa, Pohjanmaa, Finland; Vrije Universiteit Brussel, Brussels, Brussels Hoofdstedelijk Gewest, Belgium; University of Barcelona, Barcelona, Catalonia, Spain; Vrije Universiteit Brussel, Brussels, Brussels Hoofdstedelijk Gewest, Belgium

## Abstract

Civic engagement of older people remains an understudied topic in gerontological research, focusing for the biggest part on formal activities, such as volunteering and political engagement. This research proposes a more inclusive definition referring to multidimensional civic engagement which includes associational engagement, formal and informal volunteering, digital engagement, and formal and informal political engagement. Additionally, research fails to address whether older people combine different civic activities or are only engaged in one. Using EQLS data (2016) collected in 33 European countries, this research examined the multidimensional civic engagement of older people and whether they combine civic activities. Descriptive analysis was used to map the multidimensional civic engagement of those over 65 years (n = 9,031). Latent class analysis was conducted to create profiles among those who are civically engaged to study whether activities are combined and to explore the socio-structural and social capital resources of each profile (n = 6,142). The results indicate that over two-thirds of older Europeans are engaged in minimally one civic activity. Five profiles can be differentiated, ranging from low diversity profiles to a high diversity profile regarding the chance of engaging in a multitude of civic activities in later life. The results clearly show that having more resources enables diverse civic engagement in later life. This study appropriately appreciates the multidimensional civic engagement of older people and identifies the resources that must be considered when promoting multidimensional civic engagement.

